# Highly accurate classification and discovery of microbial protein-coding gene functions using FunGeneTyper: an extensible deep learning framework

**DOI:** 10.1093/bib/bbae319

**Published:** 2024-07-15

**Authors:** Guoqing Zhang, Hui Wang, Zhiguo Zhang, Lu Zhang, Guibing Guo, Jian Yang, Fajie Yuan, Feng Ju

**Affiliations:** College of Environmental and Resource Sciences, Zhejiang University, Hangzhou, Zhejiang 310058, China; Key Laboratory of Coastal Environment and Resources of Zhejiang Province, School of Engineering, Westlake University, Hangzhou, Zhejiang 310030, China; Center of Synthetic Biology and Integrated Bioengineering, Westlake University, Hangzhou, Zhejiang 310030, China; Representation Learning Laboratory, School of Engineering, Westlake University, Hangzhou, Zhejiang 310030, China; Key Laboratory of Coastal Environment and Resources of Zhejiang Province, School of Engineering, Westlake University, Hangzhou, Zhejiang 310030, China; Key Laboratory of Coastal Environment and Resources of Zhejiang Province, School of Engineering, Westlake University, Hangzhou, Zhejiang 310030, China; Software College, Northeastern University, Shenyang, Liaoning 110169, China; Westlake Laboratory of Life Sciences and Biomedicine, School of Life Sciences, Westlake University, Hangzhou, Zhejiang 310024, China; Representation Learning Laboratory, School of Engineering, Westlake University, Hangzhou, Zhejiang 310030, China; Key Laboratory of Coastal Environment and Resources of Zhejiang Province, School of Engineering, Westlake University, Hangzhou, Zhejiang 310030, China; Center of Synthetic Biology and Integrated Bioengineering, Westlake University, Hangzhou, Zhejiang 310030, China; Westlake Laboratory of Life Sciences and Biomedicine, School of Life Sciences, Westlake University, Hangzhou, Zhejiang 310024, China

**Keywords:** functional classification, protein-coding gene (PCG), deep learning, structured functional gene database (SFGD), microbiome, bioinformatics

## Abstract

High-throughput DNA sequencing technologies decode tremendous amounts of microbial protein-coding gene sequences. However, accurately assigning protein functions to novel gene sequences remain a challenge. To this end, we developed FunGeneTyper, an extensible framework with two new deep learning models (i.e., FunTrans and FunRep), structured databases, and supporting resources for achieving highly accurate (Accuracy > 0.99, F1-score > 0.97) and fine-grained classification of antibiotic resistance genes (ARGs) and virulence factor genes. Using an experimentally confirmed dataset of ARGs comprising remote homologous sequences as the test set, our framework achieves by-far-the-best performance in the discovery of new ARGs from human gut (F1-score: 0.6948), wastewater (0.6072), and soil (0.5445) microbiomes, beating the state-of-the-art bioinformatics tools and sequence alignment-based (F1-score: 0.0556–0.5065) and domain-based (F1-score: 0.2630–0.5224) annotation approaches. Furthermore, our framework is implemented as a lightweight, privacy-preserving, and plug-and-play neural network module, facilitating its versatility and accessibility to developers and users worldwide. We anticipate widespread utilization of FunGeneTyper (https://github.com/emblab-westlake/FunGeneTyper) for precise classification of protein-coding gene functions and the discovery of numerous valuable enzymes. This advancement will have a significant impact on various fields, including microbiome research, biotechnology, metagenomics, and bioinformatics.

## Graphical Abstract

**Graphical Abstract:**
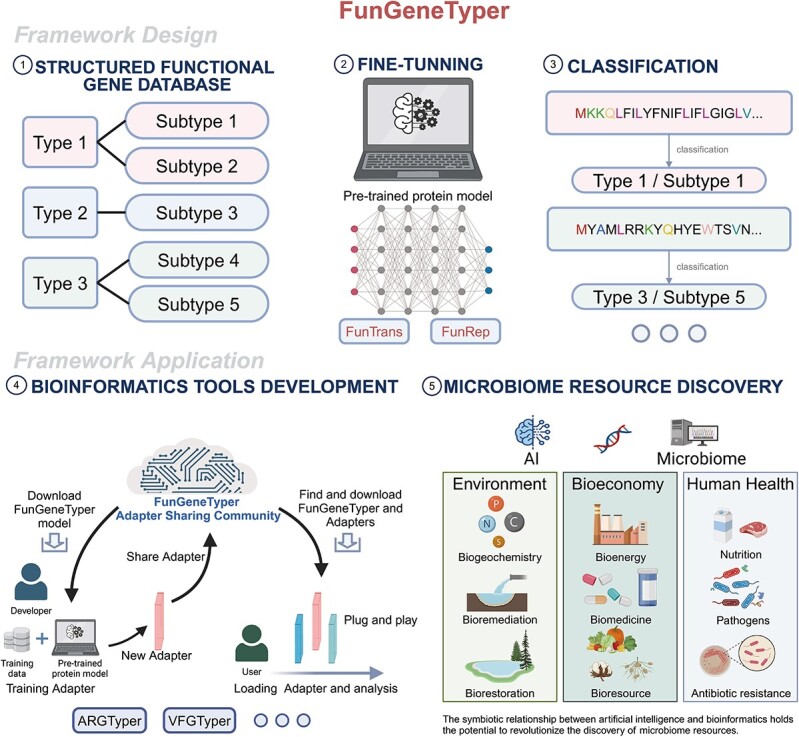

## Introduction

High-throughput DNA sequencing and metagenomics produce huge amounts of protein-coding gene (PCG) sequences from diverse environmental and human microbiomes [1–3]. Accurate functional classification of microbial PCGs is pivotal for precise comprehension and discovery of new functional genes. While several classic tools are currently available to classify PCGs to their protein families and subfamilies, analyzing these large datasets of PCGs poses computational challenges in metagenomic studies. Sequence alignment (SA), as implemented in NCBI's BLAST [4], usearch [5], and Diamond [6], is commonly used for functional annotation of PCGs [7]. This method ususally employs stringent user-defined cutoffs or thresholds, including alignment identity, coverage, and bit scores, to preserve only high-confidence and optimal matches in a reference database. For example, SA-based classification tools for functional genes, such as antibiotic resistance genes (ARGs) [8, 9] and virulence factor genes (VFGs) [10], are generally effective in clasifying the function of genes with high homology (>80% identity [8, 9]) to reference seqeuences. However, these SA-based approaches often exclude remote homologous genes that fall below arbitrarily defined and one-size-fits-all cutoffs but can account for a majority of new functional genes of interest within environmental samples (e.g. core ARGs in activated sludge [11] and soil [12]). The use of these arbitrary cutoffs results in numerous false-negative results and underestimates the true novelties (thus diversity) of functional genes in largely uncultured bacteria widespread in nature. Overcoming this bias requires developing an intelligent and precise classification paradigm, capable of surpassing the limitations of existing SA-based approaches. Such efforts are crucial to discovering new genes in future metagenomics-based microbiome studies [13, 14].

Hidden Markov model (HMM)-based tools can classify remote gene homologs with low sequence identity (<30%) to known reference proteins [15, 16]. However, such methods based on scoring matrices and E-value calculation of token (amino acid) matching fail to detect high-level semantic representation similarity or structure-level representation similarity, leading to misclassified genes [17], and thus cannot distinguish functions of proteins in the same families [18]. In contrast, deep learning (DL) methods are effective at identifying proteins with structural and functional similarities [19–22]. Ground-breaking large language models initially developed for natural language processing tasks have been successfully applied to protein function prediction tasks [23, 24]. These models, known as protein language models (PLMs), excel in learning comprehensive and sophisticated semantic representations that establish meaningful connections between gene sequences and protein function [25, 26]. However, fine-grained functional classification of PCGs poses challenges for data-hungry DL paradigms because of limited valid datasets for supervised training of function genes of interests. Additionally, the performance comparison between advanced PLMs and state-of-the-art metagenomic bioinformatics tools for microbial gene classification and discovery remain unclear.

Here, we propose and verify FunGeneTyper, a PLM-based deep learning framework for accurate and extensible prediction of PCG function. FunGeneTyper implements a two-stage pipeline that separately handles the assignment of PCGs to functional types and subtypes, reducing issues associated with insufficient training data during subtype-level predictions. FunGeneTyper first performs standard classification of PCGs to the functional types and then performs fine-grained retrieval of functional subtypes by comparing similarities between learned protein representations. FunGeneTyper models classify ARGs with high accuracy (>0.99). An ARGs dataset that were not included in the database but experimentally confirmed to confer antibiotic resistance phenotypes verified the performance of FunGeneTyper. The comprehensive performance of FunGeneTyper outperforms the state-of-the-art SA-based and HMM-based methods and tools, especially in the accurate classification of remote homologous gene sequences and the discovery of new functional genes. Furthermore, we demonstrate the generalized application of FunGeneTyper models in high-accuracy classification of VFGs and introduce the adapter module, a lightweight neural network that can be inserted into the current backbone architecture to realize parameter-efficient training. The adapter-tuning-based FunGeneTyper models are extensible to the classification of various categories of functional genes and enables sharing of both task-agnostic and task-specific parameters without accessing the private dataset. Thus, FunGeneTyper offers a unified and innovative way of integrating the global efforts of microbiome research and bioinformatics communities. Its extensible and modular design allows for unlimited prediction of functional gene categories beyond the ARGs and VFGs demonstrated here, which is key to accelerating the global discovery of new and precious genetic and enzymatic resources from microbiomes.

## Materials and methods

A complete version of the method for construct training data is available at Supplemental Information (SI).

### Architecture of the FunGeneTyper model

FunGeneTyper is a universal function classification framework composed of two core DL models, FunTrans and FunRep, which share similar structures but are designed to classify functional genes at the type and subtype levels, respectively. Both models are modular adapter-based architectures that leverage a few extra parameters to achieve efficient fine-tuning of large-scale PLMs. In detail, utilizing the state-of-the-art large-scale protein PLM ESM-1b as a 33-layer transformer encoder framework as the foundation, adapters are plugged in each transformer layer of the PLM, which are individual modular units that are used as newly introduced weights to be fine-tuned for specific functional tasks. Notably, ESM-1b, through self-supervised learning on the UniRef50 database, was shown to have a superior capacity to infer fundamental structural and functional characteristics of proteins from gene sequences [27].

**Figure 1:**
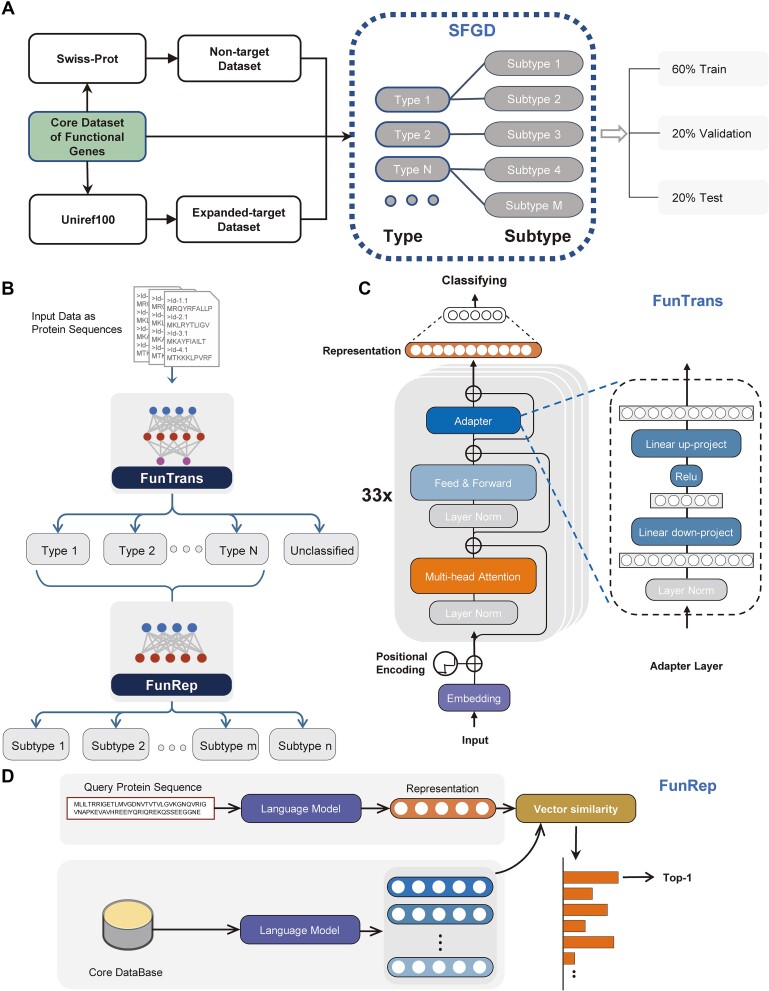
FunGeneTyper model design and database construction workflows. (A) process of preparing a SFGD. The database is divided into the training set, validation set and testing set in a 6:2:2 ratio. (B) The two-level hierarchical structure of FunGeneTyper, i.e. FunTrans and FunRep, operates in tandem, FunTrans identifies protein sequences of target function and classifies them into various types in the SFGD. The FunRep further classifies protein sequences of each type (as determined by FunTrans) down a refined level of subtype. (C) Schematic representation of FunTrans model. (D) Schematic representation of FunRep model.

The architecture is depicted in Fig. 1A and consists of three main components: a multi-headed self-attention, a feed-forward network, and an adapter layer. Each sublayer contains layer normalization and skip connections to effectively train the neural network and avoid overfitting. It is worth noting that the bottleneck-shaped adapter module consists of a down-project linear $\mathrm{H}\in{\mathbb{R}}^{d\times k}\mathrm{H}\in{\mathbb{R}}^{d\times k}$ where$d$is embedding size of the Transformer model, $k$ is the dimension of the adapter and $d\gg k$, a ReLU activation followed by an up-projection $\mathrm{L}\in{\mathbb{R}}^{k\times d}\mathrm{L}\in{\mathbb{R}}^{k\times d}$. The adapter layer is formulated as follows:


$$ {\displaystyle \begin{array}{c}{O}_l=\mathrm{LayerNorm}\left({T}_l\right)\\{}\mathrm{Adapte}{\mathrm{r}}_l={L}_l(\mathrm{ReLU}\left({H}_l\left({O}_l\right)\right)+{T}_l\end{array}} $$

where ${T}_l$ is the hidden feature at transformer layer $l$, $d=1280$, and $k=256$ in the actual training.

Following the approach of BERT [28], hidden features from the first token of the sequence of the last layer are extracted. In contrast to FunTrans, which adds a nonlinear layer for protein function classification after the representations of the last layer, FunRep first computes the hidden features of experimentally confirmed core sequences and then annotates PCGs by finding the sequence’s category with the closest Euclidean distance in the representation space.

Here, a dual-tower architecture with shared parameters similar to Sentence-BERT [29] is used for model training in order to place sequences with the same category closer in the representation space. FunRep is trained by constructing $<A,P,N>$ triples, where $A$ is the anchor sequence, $P$ is a positive example possessing the same category as $A$, and $N$ is a negative example whose category is different from $A$and the hidden representations they obtained through FunRep are $a$, $p$, and $n$, respectively. The loss function adopts Triplet Loss, which is defined as follows:


$$ \mathrm{Loss}\left(a,p,n\right)=\max \left(D\left(a,p\right)-D\left(a,n\right)\right)+\mathrm{margin},0\big) $$

where $D$ is the Euclidean distance between vectors, and $\mathrm{margin}$ is an adjustable threshold, set to 1.0 during model training. ARGTyper-FunRep and VFGTyper-FunRep are classified at the group level with the same 21.76 M learnable training parameters.

### Evaluation of FunGeneTyper for the discovery of new functional genes

The experimentally validated ARG sequences in prior functional metagenomics studies but not included in the training dataset were used to validate the FunGeneTyper model's ability to discover new functional genes (accession numbers in Dataset S8, details in Supplementary Method).

## Results

### FunGeneTyper framework, structured database, and DL models

FunGeneTyper is a unique and innovative framework that utilizes DL models and structured functional gene databases (SFGDs) to develop new DL-based classifiers, in principle, for any category of functional genes via transfer learning. This novel framework achieves highly accurate PCG classification from metagenomic studies and extends the models to efficiently predict broad categories of gene functions from large varieties of microbiomes with corresponding customizable SFGD.

#### Structured functional gene databases

We deployed a transferable strategy to collect high-quality reference protein sequences to meet FunGeneTyper's training requirements with high reliability (Fig. 1A). Experimentally confirmed reference sequences of target genes from literature and/or expert-curated databases were used as the core dataset, and highly homologous protein sequences (at least 80% identity and 80% coverage) were extracted from Uniref100 database and used as the expanded functional genes dataset. To mitigate the risk of data leakage during the training process, we implemented a precautionary measure by removing all redundant sequences that exhibited 100% identity to the core dataset. Furthermore, a nontarget sequence dataset was constructed from Swiss-Prot database by eliminating all precise matches to the target genes. The core and expanded functional gene datasets were harmoniously integrated with the nontarget dataset to form the SFGD, which was organized hierarchically into a secondary structure including type and subtypes of functional genes based on gene ontology.

#### Deep learning models

The overarching framework (Fig. 1B) is a protein functional annotation workflow named as FunGeneTyper. The framework consists of two DL models, FunTrans and FunRep, in tandem. Initially, upon receiving protein sequence data, FunTrans categorizes protein sequences into nontarget function and target function protein sequences. Simultaneously, it classifies sequences within the target function into broader categories. Subsequently, protein sequences categorized as target function are further utilized as inputs to FunRep. Through the FunRep model, these sequences undergo further functional classification, transitioning from a broader functional level (i.e., type) to a more detailed level (i.e., subtype). FunGeneTyper was pretrained on ESM-1b [22] which is composed of the 33-layer transformer architecture consisting of 650 million parameters trained on Uniref50. Both DL models of FunGeneTyper utilize the adapter architecture, facilitating the efficient fine-tuning of protein pretrained language models for diverse functional annotation tasks. Unlike the conventional approach of fine-tuning all parameters of the PLM, the adapter architecture integrates task-specific trainable modules while preserving the integrity of the underlying PLM. The adapter also enables flexible and parameter-efficient transfer learning, effectively mitigating overfitting [30, 31]. By fine-tuning just 3% of the task-specific parameter amount, we achieved superior performance, allowing for extensive parameter sharing. Furthermore, we only need to store a copy of the PLM along with a few task-specific adapter parameters instead of saving complete parameters. This approach substantially reduces storage overhead and encourages the development and deployment of a diverse protein annotation community.

FunTrans, as the initial phase of a two-stage protein annotation pipeline, is enhanced with an additional classification layer based on the architecture of adapters. It excels at performing high-throughput functional annotation of proteins at the type level (Fig. 1C). This stage effectively filters out nontargeted functional sequences and conducts a comprehensive functional classification at the type level. Subsequently, FunRep further annotates protein sequences down to a refined functional level of subtype. This is accomplished by assessing how closely the representation space aligns with the core dataset of held-out experimental validation functions (Fig. 1D). To enable the effective training of the FunRep model at the subtype level (which has a smaller number of reference sequences than the type level), we employ a contrastive learning approach which can learn more universal and distinguishable feature representations, effectively improving the generalization ability of feature representations across subtypes with different numbers of reference sequences. Since these feature representations are not overly adapted to the frequently occurring subtypes, they are generalizable to train sequences belonging to less frequent subtypes. Specifically, we randomly selected two reference sequences within the same subtype of functional genes from the core dataset, and randomly designated one as an anchor sequence and the other as a positive sequence. Meanwhile, a third sequences that do not belong to this subtype are designated as a negative sequence. For each iteration, an anchor sequence serves as the reference sequence for model learning. Each positive sequence exhibits similarity to the anchor sequence, whereas each negative sequence is dissimilar to the anchor sequence. During training, contrastive learning minimizes the distance of an anchor sequence with a positive sequence whereas maximizing its distance with a negative sequence. This is achieved by comparing the similarity between anchor and positive sequence embeddings and the difference between anchor and negative sequences embeddings, and the model iteratively improves its parameters to refine its ability to discriminate between different subtypes of protein sequences in the core dataset.

The dataset construction processes for FunTrans and FunRep are illustrated in Fig. S1. Both DL models are trained on separate adapter layers while freezing the other parameters of the protein pretrained model ESM-1b (Fig. S2). Pretrained protein models, trained through self-supervised learning on extensive, unstructured database like UniProt, generate streamlined sequence representations that capture structural, functional, and evolutionary information. Utilizing these representations for functional annotation improves the accuracy, reliability, and sensitivity of annotating understudied PCGs and acchive to rectify mislabeled functional sequences. These representations overcome the limitation that limited datasets are not conducive to unsupervised learning training. Furthermore, to address the imbalance in sample distribution among type categories in the training data, we implemented a technique of oversampling by randomly duplicating instances of the minority classes. This process ensures an equalized number of samples across different type categories within the positive dataset, thereby facilitating more effective model training and performance evaluation.

### FunGeneTyper classification performance and learning ability

**Figure 2:**
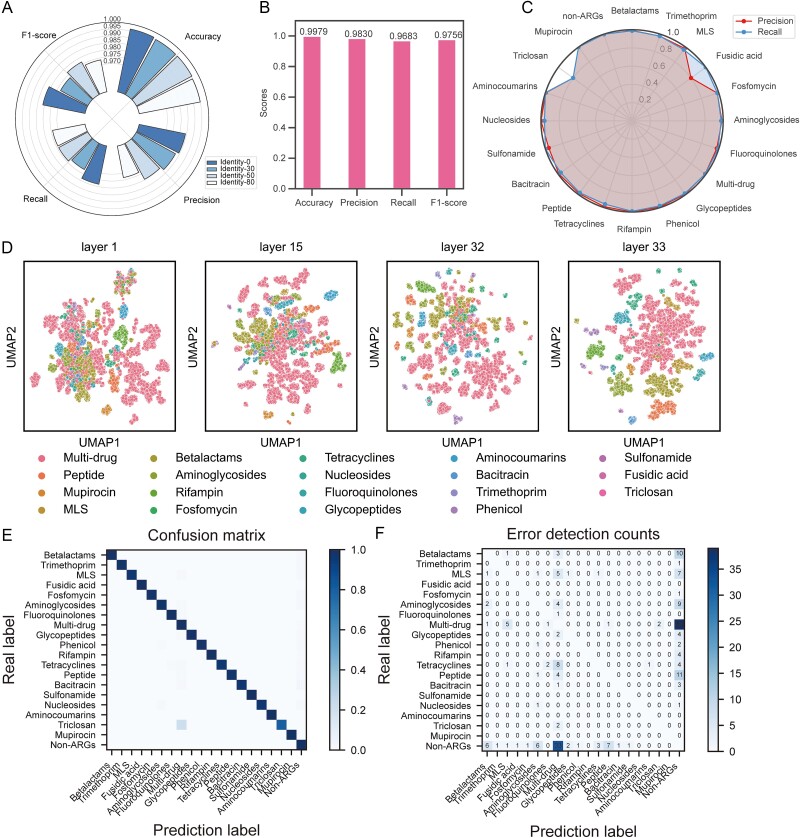
Performance evaluation of deep learning models of FunGeneTyper with Structured Antibiotic Resistance Database (SARD) for functional classification of ARGs. (A) Evaluation of the influence of identity threshold used for selecting the negative dataset on model performance in the classification of ARGs. (B) Performance metrics of ARGTyper developed based on FunGeneTyper models and SARD. (C) Classification performance of all 19 types of ARGs as indicated by precision and recall of ARGs and non-ARG classes. (D) Visualization of feature learning at different layers during the ARGTyper training process. (E) Confusion matrix for ARG type classification, confusion between true (y-axis) and predicted (x-axis) ARGs. (F) Number of ARG protein sequences annotated incorrectly. MLS: Macrolides, Lincosamides and Streptogramines.

The dissemination of antibiotic resistance poses considerable public health concerns worldwide [32]. Dependable classification of ARGs plays a vital role in surveillance and control of antibiotic resistance disseanition. Achieving adequate model sensitivity for remote homologs is the key to discovering novel ARGs. Therefore, the first application goal of this study was to classify ARGs using the FunGeneTyper framework. Before developing the ARGs classification models, we constructed the structured ARG database (SARD), a hierarchical database organized based on antibiotic resistance ontology of the comprehensive antibiotic resistance database (CARD) [7]. Utilizing CARD's ontological rules, we assigned ARG to type and subtype hierarchies based on the class of drugs against which they resist and the group of genes with the same resistance function, respectively (Dataset S1, Method S1). SARD, as a positive dataset, is used for the model to learn the important features of the target sequence. Meanwhile, negative dataset are crucial for successful training of models. To assess and enhance the model's sensitivity, we created four nontarget sequence sets from the Swiss-Prot database—excluding ARGs—as negative dataset. The datasets incoportating sequences which showed similarity to the SARD database sequences exceeding 0%, 30%, 50%, and 80% identity thresholds were removed from the Swiss-Prot database, respectively, and the remaining seqeunces were used to construct the corresponding 4 negative datasets (Fig. S3, Methods S2, S3). The addition of a negative dataset allows the model to learn features of nontargeted genes, which gives the model the ability to directly classify targeted (e.g. ARGs) and nontargeted genes (e.g. non-ARGs) from new datasets to be tested. We evaluated the impact of four identity thresholds of the negative datasets in terms of the model's feature learning. Through five-fold cross-validation, we determined that employing a 0% identity threshold for recruiting nontarget sequences resulted in superior performance metrics, including accuracy, recall, precision, and F1-score (Fig. 2A). A 0% identity threshold indicates training on a negative dataset entirely distinct from the positive dataset, prompting the model to acquire highly discriminative features for effective differentiation. This strategy facilitates the acquisition of generalizable features, mitigates overfitting, and enhances discriminative power. Subsequently, the positive dataset SARD contained 61 874 ARG sequences, including 2972 experimentally confirmed core sequences inherited from the CARD and 58 902 homology-predicted (>80% identity and >80% coverage) expanded sequences of ARGs from Uniref100. All ARG reference sequences were hierarchically assigned to 19 classes and 2972 groups (Dataset S2 and Fig. S4).

To demonstrate the powerful efficacy of FunGeneTyper, we used SARD to train the two transformer models FunTrans and FunRep, which had developed them as a DL-based classifier of ARGs named ARGTyper. The trained ARGTyper was used to classify the testing set to validate its performance in ARG classification. The overall performance metrics of ARGTyper demonstrate that FunGeneTyper is an excellent and robust framework for functional gene classification. Specifically, the optimal FunTrans model at the ARG type level exhibited an accuracy of 0.9979, a precision of 0.9830, a recall rate of 0.9683, and an F1 score of 0.9756 (Fig. 2B). To illustrate the impact of the highly similar protein sequences within the SARD on FunGeneTyper and to further validate the robustness of the model performance, we clustered the SARD sequences at the 80% sequence identity level which removed high-homology sequences and resulted in SARD80 (Method S3). Subsequently, we conducted ablation experiments to compare the performance metrics of ARGTyper with those of two other benchmark models (as described below) using the orginal SARD and SARD80. To assess the efficacy and validity of the pretrained model (ESM-1b) in FunGeneTyper, we first established a benchmark tool, denoted as ARGTyper-random, which was trained with randomly initialized parameters, devoid of any pretrained model parameters. The results demonstrated that ARGTyper (F1-score 0.9756) fine-tuned with the pretrained model outperformed ARGTyper-random (F1-score 0.6309) (Fig. S5a). These findings underscored the significance of pretrained model initialization in improving model performance. Further, we compared ARGTyper with Diamond (with parameters: --more-sensitive -e 1e-5, --max-target-seqs 1, identity >70%, Method S3), the other benchmark model based on sequence similarity. Using SARD as the training and testing datasets, ARGTyper achieved at least comparable performance to Diamond. Contrastingly, ablation results with SARD80 showed that although the removal of high-homology sequences from SARD inevitably reduced the performance of the ARGTyper (F1-score 0.8178), it achieved better comprehensive performance than the other benchmark models, i.e. ARGTyper-random (F1-score 0.2953) and Diamond (F1-score 0.7268) (Fig. S5b). These results highlight the robustness of ARGTyper, as it maintained relatively high performance even when high-homology sequences were removed. Additionally, the prediction precision and recall of all 17 ARG types exceeded 0.96 (Fig. 2C), except for fusidic acid and triclosan, which exhibited lower precision and recall because they have only 21 and 53 reference sequences, respectively, in SARD (Dataset S3). In general, although the number reference sequences in certain types and subtypes of ARGs is sparse, accurate classification performance is achieved by FunTrans model. It is essential to note that including more training data would help the model learn more features; therefore, the power of FunTrans to classify these temporarily less-represented classes of ARGs will improve as more functionally verified reference sequences will be available for model training.

FunGeneTyper produced a vector space containing comprehensive semantic information, including structural, evolutionary, and functional aspects. To provide an intuitive understanding of our model's learning ability, we obtained representations of all sequences in the training set, and reduced data dimensions in each layer using uniform manifold approximation and projection (UMAP). The learning process of the model was revealed through visualizations performed in the four essential representative layers (1st, 15th, 32nd, and 33rd) as shown in Fig. 2D. Each point represents a two-dimensional representation of the higher-dimensional features of a sequence. All ARG sequences were highly entangled at the first level of encoding input. However, they became increasingly separated as the transformer model deepened. Each type of ARG underwent a process from dispersion to aggregation. Thess findings verified that FunTrans could efficiently learn the representation features of sequences from raw input data with high entanglement.

The effect of FunTrans on the learning features of each type of ARG was represented through a prediction multiclass confusion matrix. The results indicated the excellent performance of the FunTrans model in predicting all ARG classes (Fig. 2E). We identified significant classification errors in the ARG classes using error detection counts (Fig. 2F). Prediction error was concentrated within the multidrug class, with 33 non-ARG sequences were mis-predicted as multidrug resistance, and 39 multidrug resistance protein sequences were mis-predicted as non-ARG sequences. The poor prediction performance of these proteins was mainly due to their high structural differences and diverse biological functions. These functions include roles other than multidrug resistance [33], making it challenging for a DL model to effectively learn sufficient discriminative features in the absence of sufficient training data. Multidrug efflux pumps [33] export antibiotics and other diverse extraneous substrates, including organic solvents, toxic heavy metals, and antimicrobials, and also fulfill other key biological functions such as biofilm formation, quorum sensing, and survival and pathogenicity of bacteria [33]. Therefore, multidrug resistance proteins or efflux pumps were not seriously considered as typical ARGs [17, 34]. Consequently, we recommend excluding their sequences from ARG analysis unless they can be reliably or unambiguously assigned to resistance functions of specific classes of antibiotics.

Following the demonstration of the robustness and accuracy of the FunTrans model in identifying ARGs and classifying them into 19 types, we trained FunRep to conduct more detailed lower-level classification of ARGs into 2972 subtypes (Dataset S3). FunRep achieved an overall prediction accuracy of 0.9023 for all ARG subtypes (Dataset S4). We used UMAP to visualize FunRep model's learning process except the Fusidic acid with only 21 sequences (Dataset S3). The visualization indicated that FunRep could cluster the features of each group in the major ARG types, including beta-lactams (5909 sequences), Macrolides–Lincosamides–Streptogramines (MLS, 2317 sequences), aminoglycosides (3483 sequences), and glycopeptides (2037 sequences) (Fig. S6).

In summary, our study first demonstrated the power of ARGTyper, the first transformer-based ARG classifier of its kind developed using the FunGeneTyper framework. The performance metrics of the testing set demonstrated that FunTrans and FunRep could accurately (accuracy = 0.998) and robustly (F1-score = 0.976) identify all known types (classes) and subtypes (groups) of ARGs in the authoritative CARD. Notably, both the accuracy and robustness of FunGeneTyper models outperform previously published results from DeepARG (accuracy > 0.97, F1-score > 0.93) [9] and HMD-ARG (accuracy = 0.935, F1-score = 0.893) [35] on their own testing sets of ARGs.

### Model performance in the discovery of novel genes

The 'twilight zone' of protein sequence alignment (SA) is a long-standing and intricate problem that hinders protein function prediction [36, 37] and limits the discovery of functional genes from the largely uncultured microbes or microbial dark matter. In contrast to classic SA-based tools, the DL-based models (FunRep and FunTrans) of the FunGeneTyper framework are designed with unique features and intrinsic advantages for accurately and robustly predicting remote homologs of protein sequences. This capability has been above-demonstrated for ARG classification.

To compare FunGeneTyper's ability to identify new functional genes with those of existing methodologies, we evaluated the ability of its DL-based models to discover remote homologs by predicting experimentally confirmed protein sequences of ARGs newly discovered from three representative habitats: human gut (*n* = 168) [38], wastewater treatment plants (WWTPs) (*n* = 77) [11], and soil (*n* = 52) [39–42]. We computed the predictive performance of FunGeneTyper classifier for ARGs (ARGTyper) and compared it with that of three state-of-the-art tools (Method S4): DL-based tools (HMD-ARG [35] and DeepARG [9]), alignment-based tools (RGI [7]), and HMM-based tools (Resfams [18]) (Table 1). Both DeepARG and HMD-ARG utilized their original training models to compare the performance of each tool under real-world test conditions. Overall, the results showed that FunGeneTyper had higher accuracy, precision, recall, and F1-score for predicting new ARGs compared with HMD-ARG [35] and DeepARG [9]. The significant improvement was primarily attributed to our implementation of the protein semantic models (i.e. FunTrans and FunRep) in FunGeneTyper, which can learn more hidden features of protein sequences, especially the context information [19, 21], compared to the traditional one-hot encoding algorithm and the convolutional neural network used by HMD-ARG [35] and the multilayer perceptron used by DeepARG [9]. Moreover, the overall classification performance of FunGeneTyper, as benchmarked by the F1-score (0.5445–0.6948), was much higher than that of the classic SA-based methods (0.0556–0.6598) and HMM-based methods (0.2630–0.5224) (Table 1). Although RGI also achieved high accuracy (0.8830) in human intestinal data, its precision (0.4545), recall (0.3968), and F1-score (0.4195) were much lower than those of the FunTrans model (0.7500, 0.6642, and 0.6948, respectively) because many of the new ARG sequences tested here fell below the commonly applied stringent identity cutoffs (>95% RGI). Thus, applying a strict one-size-fits-all cutoff to filter the alignment results is likely to result in many false-negative results, limiting the discovery of ARGs showing more remote homology to database sequences. Comparative tests conducted using WWTP or soil samples compared with human gut samples (Table 1) generally demonstrate FunGeneTyper's superior performance to predict functional genes in complex environmental samples. To further resolve the superior predictive performance of FunGeneTyper for remote homologs of functional genes over existing tools, we divided the ARG sequences into lower homology (≤50% identity) and higher homology (≥50% identity) datasets according to the amino acid identity of experimentally confirmed protein sequences with those core dataset of ARGs (Fig. S7). FunGeneTyper not only consistently achieved better classification performance of higher homology ARGs in all three sample groups (WWTP, soil, and human gut), but also showed outstanding performance in accurate and sensitive functional prediction of remote homologous sequences (Dataset S5).

**Table 1:** Performance comparison between FunGeneTyper and other alternative bioinformatics tools for the discovery of experimentally confirmed new ARGs. In total, 297 experimentally confirmed ARGs sequences of human gut [38] (*n* = 168), WWTPs [11] (*n* = 77), and soil [39–42] (*n* = 52) bacteria were included in the comparative analysis which was performed under the default settings of each deep learning (DL)-based, sequence alignment-based or hidden Markov model (HMM)-based tool recommended by the developers.

**Tools**	**Human gut (*n* = 168)**				**WWTP (*n* = 77)**				**Soil (*n* = 52)**			
	**Accuracy**	**Precision**	**Recall**	**F1-score**	**Accuracy**	**Precision**	**Recall**	**F1-score**	**Accuracy**	**Precision**	**Recall**	**F1-score**
*DL-based tools*												
FunGeneTyper	0.8512	**0.7500**	**0.6642**	**0.6948**	**0.7273**	**0.7500**	**0.5403**	**0.6072**	**0.8269**	**0.5926**	**0.5529**	**0.5445**
HMD-ARG	0.8452	0.6000	0.5230	0.5486	0.5714	0.7161	0.3877	0.4589	0.8077	**0.6000**	0.4560	0.5119
DeepARG	0.3512	0.6250	0.4720	0.5149	0.1688	0.5714	0.1682	0.2591	0.2885	0.3750	0.1057	0.1607
*Alignment-based tools*												
RGI	0.3452	0.6250	0.4596	0.5065	0.0390	0.3750	0.0349	0.0632	0.1538	0.1250	0.0357	0.0556
*HMM-based tools*												
Resfams	**0.8830**	0.4545	0.3968	0.4195	0.6234	0.6250	0.4736	0.5224	0.8088	0.2727	0.2545	0.2630

Bold indicates the highest value in each column.

Taken together, our study demonstrates FunGeneTyper’s exceptional ability to predict novel ARG protein sequences with unparalleled accuracy, sensitivity, and robustness. Our results support the discovery and classification of novel ARGs, especially among relatively remote homologs with less than 50% identity.

### Evaluating the generalizability of FunGeneTyper

To demonstrate the generalizability of FunTrans and FunRep in classifying other categories of functional genes, we trained a new transformer-based classifier of VFGs, named VFGTyper, using a calibrated and professionally expanded bacterial virulence factor database (VFNet) [43]. The database was meticulously cleaned to remove semantic and categorically ambiguous data (Method S5). The final structured virulence factor database (SVFD) comprised of 160 484 VFG sequences which were distributed into 2837 classes in 45 families (Dataset S6).

**Figure 3:**
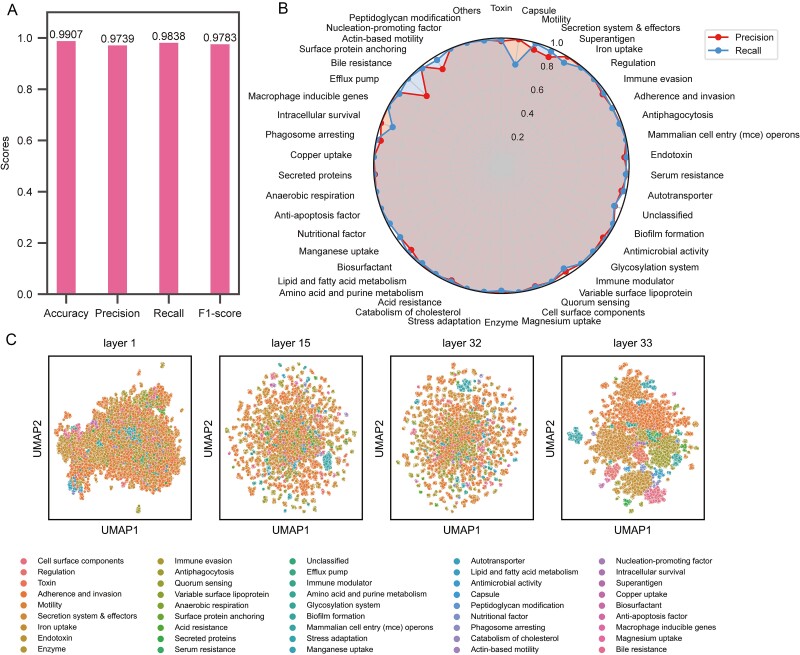
Transfer learning of FunGeneTyper models on structured virulence factor gene database (VFGD) and performance evaluation for VFG classification. (a) Performance metrics of VFGTyper developed based on FunGeneTyper models and VFGD. (b) Precision and recall of VFGs family and non-VFGs category. c, visualization of feature learning at different layers in VFGs FunTrans training. VFGs: Virulence factor genes.

The design of the adapter model allowed us to selectively retrain only a new adapter when developing VFGTyper. This adapter module facilitated training of a new classifier and adapter while utilizing the preexisting parameters in the backbone network. Consequently, VFGTyper can be considered as a distinct task branch within the FunGeneTyper, distinguished solely by the adapter and classifier components. We verified the VFGTyper using the testing set to provide evidence of its generalizability in the highly accurate classification of VFGs. VFGTyper achieved an accuracy of 0.9907 (Fig. 3A) in the family level prediction task. The obfuscation matrix results also showed that FunTrans achieved excellent classification performance for each VFG at the family level (Fig. 3B, Fig. S8). In addition, FunRep achieved an accuracy of 0.9499 at predicting different VFG classes in the second-stage prediction. For original SVFD datasets, VFGTyper (F1-score 0.9783) performed better than the VFGTyper-random (F1-score 0.6341) benchmark model (Fig. S9a). The results of the sequence ablation experiment also indicated that VFGTyper (F1-score 0.8402) performed better than the benchmark models VFGTyper-random (F1-score 0.3714) and Diamond (F1-score 0.6187) (Fig. S9b).

In conclusion, we demonstrated that FunGeneTyper can be extended to generate VFGTyper that achieves highly accurate VFGs classification by introducing new adapters. Consistent with the learning process for ARGs (Fig. 2D), both models also achieved effective feature clustering and classification of VFGs at both the family (Fig. 3C) and class (Fig. S10) levels. Besides classification performance, we also proved VFGTyper's full capability in the discovery of an experimentally confirmed novel VFG (NCBI accession no.: WP_034687872.1) of a toxin family in *Chryseobacterium piperi* with sequence similarity to botulinum neurotoxins (BoNTs) through re-analysis of published genomes [44]. Specifically, of the eight putative toxin genes of *C. piperi* showing no significant (*n* = 6) or only limited (*n* = 2) sequence homology (i.e. global identity <10%) to known reference VFGs, seven were effectively identified as VFGs by FunGeneTyper and four were further classified as BoNTs (Dataset S7). Compared to the conventional SA-based approach which failed to predict six VFGs, the DL models of FunGeneTyper demonstrated much greater capacity for the discovery of remote homologs of known toxin genes. Therefore, FunGeneTyper represents an extensible DL-based framework that is scalable for the highly accurate classification and discovery of protein functions, as demonstrated here for ARGs and VFGs.

### Privacy-preserving global sharing of plug-and-play adapters for functional gene discovery

To demonstrate the parameter efficiency of FunGeneTyper's adapter modules, all 650 million parameters of the pretrained model are fine-tuned as a benchmark test which achieved excellent prediction accuracy in ARGs type (0.9988) and VFGs family (0.9930). Comparatively, with only fine-tuning of about 21 million parameters (3% of all parameters) of the adapter layer, we demonstrated that FunGeneTyper achieved near-identical excellent performance of 0.9979 for ARGs class and 0.9907 for VFGs family, proving that parameter-efficient lightweight plug-and-play adapter modules of FunGeneTyper can be easily shared with little loss of prediction accuracy.

**Figure 4:**
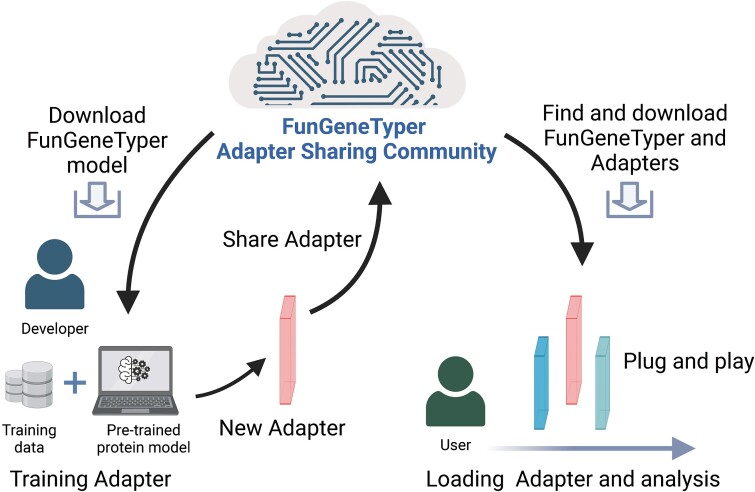
Schematic of the Adapter Sharing Community (ASC) in the framework of FunGeneTyper. The community developers are cyber de-centralized to train customizable structured databases and develop deep learning classifiers of various categories of functional genes, while users utilize the classifiers of interest to accelerate the discovery of genes which, in turn, provide new experimentally confirmed sequences to expand the structured databases and improve deep learning models.

The parameter-efficient nature of FunGeneTyper means it has two novel merits. First, FunGeneTyper enables effective effort-sharing by the entire community (Fig. 4). Specifically, researchers who has trained our FunGeneTyper model for classification or discovery of protein functions (other than ARGs and VFGs demonstrated here) can submit their adapters (along with a classification layer) to the adapter hub. Once the adapter has been submitted, the module can be downloaded and easily inserted into the FunGeneTyper model for direct application by downstream users. Second, researchers who have not publicly released their own datasets can protect their private datasets by train FunGeneTyper with the data and submitting only the adapter module (again along with a classification layer) and providing functional descriptions of their FunGeneTyper. As a result, the private datasets remain protected, while the uploaded adapter models can be used without model training. The model may become a universal toolkit that can be used for predicting functional genes simply by looking up related functional modules. With the elegant adapter module, FunGeneTyper enables efficient adapter sharing and model integration globally, thereby promotes bioinformatics development in the fields of computational biology, microbiome, and metagenomics.

### Usage of computer resources

The operational efficiency of FunGeneTyper was tested using a subset of 1000 protein-coding gene sequences. The ARGs classification task was ran at supercomputer center. When using 1 GPU (NVIDIA A40, 48 Gb), the elapsed time is about 46 s. Comparatively, about 75 min 46 s when using 1 core of CPU (AMD 7713, 2.0 GHz). Considering that deep learning uses graphics cards to accelerate computation, we recommend using GPU servers to accelerate classification and prediction tasks of functional genes.

## Discussion

Metagenomics presents an opportunity for identifying genetic diversity and novel functionalities from microbiome, especially uncultured microbes. However, the speed at which high-throughput DNA sequencing technologies unravel the vast genetic novelties of microbes far outpaces our capacity to understand their function. Previous approaches for functional classification of genes were based on SA using tools such as BLAST [4], usearch [5], and Diamond [6] or conserved motifs and domains using HMMs. However, these approaches have limitations in classifying functional genes, primarily due to uniform cutoffs applied for filtering alignment results. Protein semantic algorithms based on natural language processing (NLP) methods have also been developed [20, 24]. However, these algorithms are not optimized for classifying specific microbial genes, and a unified paradigm is required to meet the demands of rapidly discovering new genes.

Our study provides an extensible DL-based end-to-end FunGeneTyper framework that facilitates efficient and robust protein-coding gene function prediction. This framework represents an emerging methodological paradigm for global developers and users to tackle unprecedented challenges and meet the above-mentioned urgent needs in the classification and discovery of diverse groups of functional genes. As a proof of concept, we implemented the framework by developing two transformer-based classifiers, ARGTyper and VFGTyper, that utilize DL models coupled with expert-curated structured databases (SARD and SVFD). These new classifiers enable robust functional classification of bacterial ARGs and VFGs, which are two categories of functional genes key to WHO's one health approach for human, animal, and environmental health protection [45].

Our study presents a series of experimental validations, including five-fold cross-validation, testing set validation, and experimentally confirmed protein sequence validation, and demonstrates the effectiveness and robustness of FunGeneTyper. Using ARG as an example, we use experimentally verified ARGs datasets with confirmed resistance phenotype as a benchmark to prove that FunGeneTyper models are more effective than SA-based and DL-based models in predicting functionally validated protein sequences of new ARGs (not included in the database) from the human gut, WWTP, and soil microbiomes with relatively low homology (< 50% similarity) to known ARGs. The superior performance of ARGTyper classifier indicates that the learning capability of the protein semantic models implemented in our FunGeneTyper framework can efficiently discover new functional genes. Given that experimentally confirmed sequences are not always sufficient, expanding the database based on sequence homology is common and necessary to acquire adequate training data. UMAP analysis demonstrated the reliability of the expanded sequences. This expansion enables our model to effectively learn discriminative protein semantic features, leading to satisfactory performance in identifying functional genes.

Accurately classifying target genes amidst the vast amount of nontarget gene data presents a challenge. Therefore, we purposefully introduced nonfunctional genetic datasets as part of the training set. This operation enables our model to accurately classify target genes from amidst noisy data from different microbiome samples. Some machine learning methods rely on SA tools to create a similarity score matrix of potential gene sequences and databases [9, 46]. Such practices will inevitably be affected (and limited) by the selection of arbitrary thresholds for the results. The FunGeneTyper framework proposed here can accurately classify genes through discriminative features learned from multiple sequences. The limited number of training sequences may prevent the models from learning sufficient features. However, this issue would be easily resolved when more experimentally confirmed reference protein sequences of target genes become available for model retraining and refinement. Furthermore, the robustness of DL to noisy labels [47] can also enhance the performance of our framework models and classifiers in discovering novel genes compared to existing approaches.

**Figure 5:**
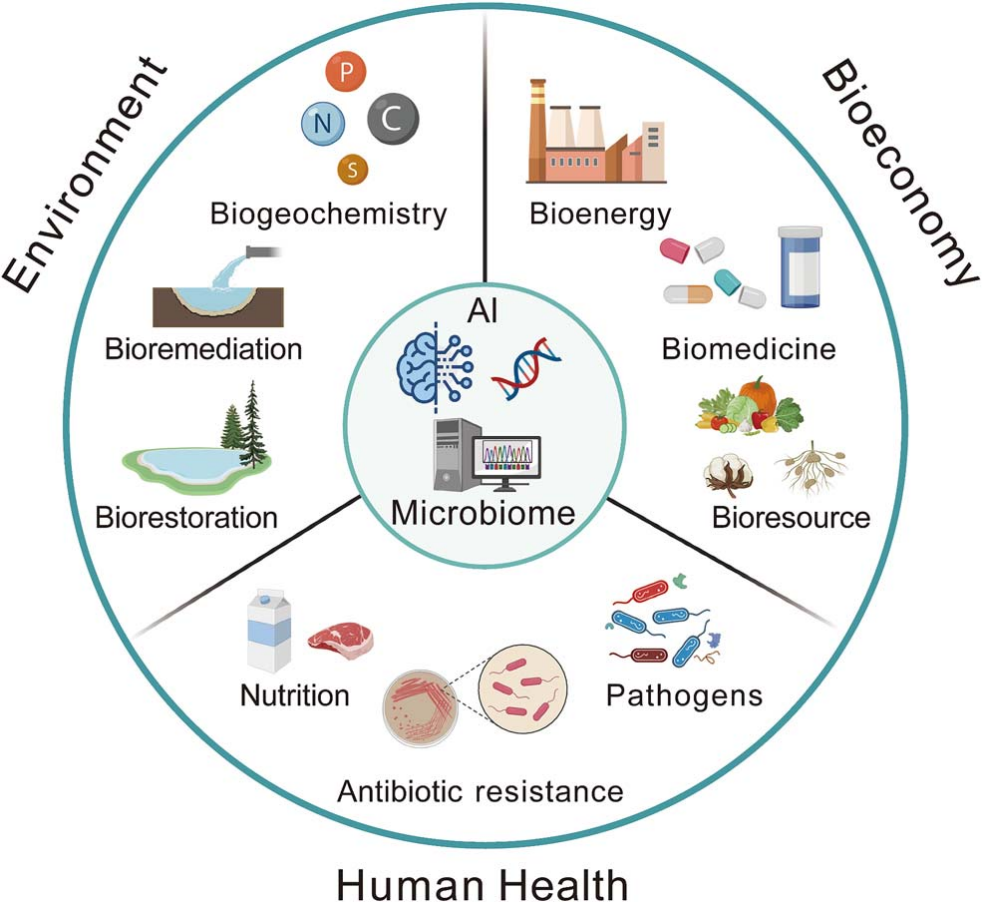
Potential applications of FunGeneTyper to accelerate the discovery of microbiome resources for enhancing our environment, bioeconomy, and human systems. Metagenomic discovery of precious genetic and enzymatic resources facilitated by the adapter sharing community of FunGeneTyper can contribute to follow-up microbiome, genetic and protein engineering research for enhancing human health and eco-environment systems.

With the rapidly accumulating microbial (meta)genomic data deposited in the global public databases—such as NCBI’s Sequence Read Archive (SRA), European Nucleotide Archive (ENA), and China National GeneBank DataBase (CNGBdb)—establishing a comprehensive understanding of the mapping relationship between microbial gene sequences and protein function poses a perennial challenge, yet offers significant prospects for accelerating the discovery of valuable genetic and enzymatic resources from various microbiomes especially for uncultured microbes therein. Researchers recently presented a machine learning model CLEAN [48] to successfully predict 36 promising biocatalytic enzyme-coding genes experimentally confirmed to involve in carbon-halogen bond formation. This subsequent milestone progress play coincides with that demonstrated earlier by FunGeneTyper [49] built on exactly the same pretrained language model ESM-1b, jointly showcasing the efficacy and unprecedent performance of contrastive learning in predicting the functions of uncharacterized proteins and under-studied enzymes. One step further, FunGeneTyper offers a broader application scope by integrating lightweight, privacy-preserving, and plug-and-play neural network modules sharable among global developers and users. This advanced design empowers FunGeneTyper as a superior DL-based framework easily extensible to the discovery of other categories of functional genes other than ARGs and VFGs demonstrated here, such as those deposited and well classified in the RDP's FunGene database [50], promoting exploration of their functional roles in promoting our environment, bioeconomy, and human health (Fig. 5). Establishing a dynamic metagenomic bioinformatics community can improve our understanding of gene function. The integration of artificial intelligence techniques with bioinformatics, as exemplified by FunGeneTyper, holds tremendous potential in advancing our understanding of gene function as one of the next frontiers of microbiome research. FunGeneTyper's adaptability is particularly noteworthy, as it can predict the function of various gene categories by PLMs and fine-tuned adapter model. The adapter module used in FunGeneTyper is a lightweight plug-and-play neural network that only fine-tunes and maintains a small set of parameters and is conducive for sharing and promotion. Crucially, the establishment of a dynamic community of metagenomics and microbial bioinformatics, guided and interconnected by the framework of FunGeneTyper, fosters collaboration and knowledge sharing among global researchers. Through the sharing of training parameters and adapters via the adapter sharing community (ASC), scientists can easily develop predictive DL models of functional genes tailored to their specific research interests without disclosing proprietary datasets. The thriving and collaborative ASC, guided by the FunGeneTyper framework, provide a dynamic, interactive, and continuously improving or evolving platform for functional classification of various gene sequences. This collaborative ecosystem fueled by FunGeneTyper not only enhances the accuracy of gene classification but also accelerates the discovery of novel enzymes and proteins with diverse applications. More importantly, FunGeneTyper empowered by ASC is expected to contribute significantly to the highly accurate prediction of protein functions as well as the discovery of valuable enzymes that advances many fields, such as industrial biotechnology, health and medicine, food and agriculture, environmental biotechnology, and bioenergy (Fig. 5). As future researchers increasingly adopt artificial intelligence-powered tools like FunGeneTyper to explore microbiomes and uncultured microbes, the pace of new protein and enzyme-coding gene discovery is expected to prominently accelerate, driving technological innovations promoting future bioeconomy as well as addressing key challenges in environmental and human systems. Thus, the symbiotic relationship between artificial intelligence and bioinformatics, facilitated by FunGeneTyper and ASC, promises to revolutionize microbiome resources discovery and unlock their biotechnological potential for next-generation bioeconomy.

In conclusion, FunGeneTyper provides an innovative and unified framework with DL models (i.e. FunTrans and FunRep), extensible classifier toolkits (e.g. ARGTyper and VFGTyper), and customizable structured databases (e.g., SARD and SVFD) for the highly accurate classification and discovery of protein functions (e.g. ARGs and VFGs) that have scientific and biotechnological significance. The framework will significantly advance the highly accurate surveillance of health-related protein functions (e.g. bacterial antibiotic resistance and virulence demonstrated in this study) as well as promote the discovery of uncharacterized but valuable enzymes. Such endeavors are in turn critical to understanding and harnessing the microbiome sciences and bioresources from our environment (biogeochemistry, bio-restoration, and bioremediation) [14], bioeconomy (bioenergy and bioresources) [13], and human systems (food and health) [20, 51].

**Key Points**

- Accurately assigning protein functions to new gene sequences remains challenging, especially for the discovery of novel functional gene sequences with low homology to known ones.
- We developed an end-to-end FunGeneTyper framework, an innovative and extensible DL-based framework with novel models, structured databases, and new bioinformatics tools for highly accurate and fine-grained classification and discovery of functional protein-coding genes.
- The paradigm and framework can be utilized to develop new plug-and-play neural network lightweight adapters and enable the establishment of an Adapter Sharing Community (ASC).
- The FunGeneTyper and ASC will be widely used to accurately classify protein functions as well as discover numerous valuable enzymes from microbial dark matter, thereby advancing many fields, such as microbiome, biotechnology, and bioinformatics.

## Supplementary Material

Supplementary_Information-2BiB-Short-final-proof_bbae319

Dataset_bbae319

## Data Availability

The code and training data underlying this article are available in GitHub (https://github.com/emblab-westlake/FunGeneTyper).

## Abbreviations

ARG: Antibiotic Resistance Gene

BoNTs: Botulinum NeuroToxins

DL: Deep Learning

MLS: Macrolides, Lincosamides, and Streptogramines

PCG: Protein-Coding Gene

PLM: protein-language model

SFGD: Structured Functional Gene Database

VFG: Virulence Factor Gene

## Author Biographies

**Guoqing Zhang** is a PhD student in the College of Environmental & Resources Science, Zhejiang University and the School of Engineering, Westlake University. His research interests are deep learning, bioinformatics and antibiotic resistome.

**Hui Wang** is an exchange student in the School of Engineering, Westlake University. His research interests are deep learning, protein language model.

**Zhiguo Zhang** is a PhD student in School of Engineering, Westlake University. His research interests are antibiotic resistome and bioinformatics.

**Lu Zhang** is a postdoctral researcher in the School of Engineering, Westlake University. Her research interests include environmental science and antibiotic resistome.

**Guibing Guo** is a professor in Software College, Northeastern University. His research interests include recommender systems, deep learning, social network analysis and data mining.

**Jian Yang** is a professor at the Westlake Laboratory of Life Sciences and Biomedicine, and the School of Life Sciences, Westlake University. His primary research interests focus on understanding the genomic variations among individuals within and between human populations, as well as the associations of DNA variations and modifications with complex traits and diseases.

**Fajie Yuan** is an assistant professor in the School of Engineering, Westlake University. His research interests are deep learning, recommender Systems.

**Feng Ju** is an associate professor at the Environmental Microbiome and Biotechnology Laboratory (EMBLab: www.ju-emblab.com), School of Engineering, and the Westlake Laboratory of Life Sciences and Biomedicine, School of Life Sciences, Westlake University. His primary research interests focus on understanding the animal and environmental microbiome structure and function, as well as the nexus among microbiome, bioeconomy and human health.
