# Supplementary material for: Highly accurate classification and discovery of microbial protein-coding gene functions using FunGeneTyper: an extensible deep learning framework
Source: Brief Bioinform. 2024 Jul 15;25(4):bbae319. doi: 10.1093/bib/bbae319 (PMC11247404; doi:10.1093/bib/bbae319)
Supplement: Supplementary_Information-2BiB-Short-final-proof_bbae319 [file supplementary_information-2bib-short-final-proof_bbae319.docx]

**Supporting Information for**

Highly-Accurate Classification and Discovery of Microbial Protein Functions Using FunGeneTyper: An Extensible Deep Learning Framework

* Correspondence to Dr. Feng Ju and Dr. Fajie Yuan

Email: jufeng@westlake.edu.cn and yuanfajie@westlake.edu.cn

**This PDF file includes:**

Supplementary Method

**Method S1.** Collection and expansion of the core dataset

**Method S2.** Construction of structured antibiotic resistance database (SARD)

**Method S3.** Training settings

**Method S4.** Evaluation of FunGeneTyper for the discovery of new functional genes

**Method S5.** Construction of structured virulence factor database (SVFD)

Supplementary Figure 1 to 10

**Figure S1.** Diagram of dataset construction for FunTrans and FunRep.

**Figure S2.** Diagrammatic representation of FunTrans and FunRep training architectures**.**

**Figure S3.** The identity threshold for negative dataset expansion.

**Figure S4.** The number of ARGs class sequences in the core SARD.

**Figure S5.** Baseline testing and sequence similarity ablation analysis of SARD.

**Figure S6.** Visualization of feature learning at the layer 33 in ARGs FunRep training. Each point is a two-dimensional mapping of a sequence of higher-dimensional representations.

**Figure S7.** An overview of the alignment of experimentally confirmed protein sequences.

**Figure S8.** Confusion matrix for VFs family level annotations.

**Figure S9.** Baseline testing and sequence similarity ablation analysis of SVFD.

**Figure S10.** Visualization of feature learning at the layer 33 in FunRep training of VFs.

Legends for Datasets S1 to S8

**Dataset S1.** The details of ARGs in the core dataset of Structured Antibiotic Resistance Database (SARD). The core dataset of SARD is originated from the reference protein sequences of ARGs defined in the authoritative comprehensive antibiotic resistance database (CARD). The naming of Gene family, Subclass and Group is according to the Antibiotic Resistance Ontology (ARO) information in the CARD was manually inspected.

**Dataset S2.** The details of ARGs in the core and expanded datasets of Structured Antibiotic Resistance Database (SARD). The SARD includes 2972 core ARGs from CARD and 58902 expanded ARGs from Uniref100.

**Dataset S3.** The count statistics of ARGs class and group in the Structured Antibiotic Resistance Database (SARD). The ARGs were classified into 20 classes and 2972 groups.

**Dataset S4.** The classification performance of ARGTyper on the test set of ARGs from Structured Antibiotic Resistance Database (SARD).

**Dataset S5.** Comparison of classification results in experimentally-confirmed protein sequences. FunGeneTyper models showed outstanding performance in predicting the function of remote homologous sequences (identity < 50%) with greater accuracy and sensitivity than existing tools.

**Dataset S6.** The count statistics of family and class of virulence factor genes (VFGs) in the structure virulence factor dataset (SVFD). The VFGs in SVFD were classified into 45 families and 2837 classes.

**Dataset S7.** FunGeneTyper showed greater capacity for the discovery of remote homologues of known toxin genes compared with sequence alignment (SA)-based approach. Among the 8 putative toxin genes of C. piperi showing no significant (n=6) or only limited (n=2) sequence homology (i.e., global identity < 10%) to matched reference VFGs in SVFD, 7 were effectively identified as VFGs by FunGeneTyper and 4 were further classified as BoNTs. However, the conventional sequence alignment (SA)-based approach based on Diamond research failed to predict 6 of the 8 putative VFGs.

**Dataset S8.** The list of experimentally confirmed protein sequences from Human Gut, WWTP and soil samples. This dataset was downloaded from the NCBI's database according to the Accession number, and those new ARG sequences not included in the reference database were used for comparative performance evaluation of FunGeneTyper with other bioinformatics tools in the discovery of new ARGs.

**Other supporting materials for this manuscript include the following:**

Datasets S1 to S8

**Supplementary Method**

**Method S1. Collection and expansion of the core dataset**

The core dataset used for FunGeneTyper model training is a set of experimentally-confirmed reference sequences of target functional genes collected from literature and/or expert-curated databases. Because the core dataset does not always contain a sufficient number of experimentally confirmed sequences (no more than 10 sequences[1]) for every type or subtype of functional gene, it is expanded to retrieve more sequence data to improve and optimize the training of deep learning models. In the subsequent training method, which separates the extended categories of five or more sequences into the training set, verification set, and testing set at a ratio of 6:2:2, any categories that are unsuitable for inclusion in these five sequences are included in the training set.

**Method S2. Construction of structured antibiotic resistance database (SARD)**

*Core ARGs dataset.* To ensure the professionalism and accuracy of the training dataset, reference protein sequences of ARGs defined by homologs in the authoritative CARD (version 3.1.2, download in June 2021) were selected as core data for downstream model training. The sequences were clustered using CD-HIT[2] (v4.8.1) at an amino acid sequence identity of 100%, and all protein sequences and their ontological information were manually checked to ensure that each ARG was properly classified into class (type) and group (subtype) based on their ontological information. Generally, class is equivalent to CARD’s ontology terms for antibiotic drug types, and group is equivalent to the specific sequence category. Macrolides, lincosamides, and streptogramins were combined into the MLS class. Based on the above procedures, a core dataset of 2972 non-redundant sequences representing 2972 groups of ARGs from 19 classes was obtained and used to build the SARD, which was used in subsequent analyses.

*Expanded ARGs dataset.* To ensure sufficient training data, the core dataset was expanded by retrieving close homologues of its ARGs from the Uniref100 database (download in June 2021) following strict screening criteria. Briefly, Diamond[3] (version 2.0.15) was used to index the ARG sequences in the core dataset and to search for homologous sequences with an amino acid identity and coverage greater than or equal to 80%. Remove all redundant sequences that have 100% identity to the core dataset. The extracted candidate sequences were dereplicated and used as expanded datasets.

*Negative dataset.* To ensure that the model can learn sufficient features of non-target gene function, which is essential for robustly predicting target function directly from metagenomic data, we used the Swiss-Prot database, an expert-validated protein database, to generate a negative dataset for use as a non-ARG training set. First, protein sequences associated with antibiotic resistance in the Swiss-Prot database (version: June 2021) were screened out using the keywords KW-0046. The remaining sequences were aligned against the core ARGs dataset using Diamond software. Sequences with an alignment coverage greater than 80% were extracted and categorized into four negative datasets based on their sequence alignment identity (ID): identity 0 (ID ≤0%, sequence counts: 453,481), identity 30 (ID ≤30%, sequence counts: 470,358), identity 50 (ID ≤50%, sequence counts: 474,570), and identity 80 (ID ≤80%, sequence counts: 475,049).

**Method S3. Training settings**

*FunTrans training*

*Dataset Construction:* The training set constructed by applying four negative dataset threshold conditions using 5-fold cross-validation method is trained to select the optimal negative dataset threshold scheme. The negative dataset with an identity threshold of 0% is selected for training the final model.

*Training details*: In the final model training, all datasets are divided into training, validation, and testing in a 6:2:2 ratio. To tackle the significant imbalance in sample quantities among functional sequence categories, where some categories contain only a few sequences while others have thousands, we implement oversampling techniques during model training. More specifically, we balance the sample distribution among type categories in the training data by randomly duplicating instances of the minority classes to equalize the number of samples across different type categories within the positives. For instance, in Type-level functional classification, the sample counts for Type1, Type2, Type3, and the Negative Category are 30,000, 5,000, 500, and 200,000, respectively. Through oversampling, we adjusted the counts to 30,000 for every positive category, while leaving the Negative Category unchanged. It is important to note that the number of non-functional category samples still surpasses the number of functional category samples. This observation reflects real-world conditions and partially validates the model’s feature extraction capabilities.

*FunRep training*

*Constructing dataset:* We divide the Expanded functional genes dataset into training, validation, and testing sets in a 6:2:2 ratio according to subtype, with a small amount of high-quality Core functional data allocated to the training set.

Training details: Contrastive learning is utilized for model training by establishing triplet datasets consisting of anchor, positive, and negative sequences. Specifically, two sequences within a subtype are randomly chosen as the anchor and positive examples. For the negative sequence, 50% are selected from the same type but different subtype, and the other 50% from a different type of category. This approach enhances the model’s ability to discern distinctive features of each subtype. During validation and testing, each sequence in the validation and test sets is annotated by comparing vector similarities with all sequence in the core dataset, assigning the subtype category of the most similar sequence for annotation. Euclidean distance is employed to quantify the similarity between vectors.

In our experiment, each group and class of ARG and VFG were padding with 21,217 and 27,535, respectively. Adam optimizer with default parameters is used, dropout is set to 0.2, learning rate is 1e-5, and the early stopping method is adopted to prevent overfitting. The accuracy, precision, recall and F1-score are used to evaluate the performance. As a result, the micro average of the F1-score also equals that of precision and recall, as well as the overall accuracy. Thus, we report only the overall accuracy for the micro average metrics while reporting precision, recall and F1-score for the macro average metrics. In this study, four evaluation metrics including the accuracy, precision, recall and F1-score were computed to assess the multi-classification results performance using the following equations:

$$Accuracy= \frac{TP+TN}{TP+FP+FN+TN}$$

$$Precision= \frac{TP}{TP+FP}$$

$$Recall= \frac{TP}{TP+FN}$$

$$F1 Score= \frac{2* Precision*Recall}{Precision+Recall}$$

where$\mathrm{TP}$ is the number of true positives, $\mathrm{TN}$ is the number of true negatives, $\mathrm{FP}$ is the number of false positives, and $\mathrm{FN}$ is the number of false negatives.

Baseline test

The baseline test datasets were the original SARD and SVFD and the SARD80 and SVFD80 datasets using the CD-HIT[2] tool to remove redundant sequences based on 80% sequence identity from the SARD and SVFD. The dataset is partitioned into training validation and testing set in a ratio of 6:2:2. Consequently, the sequences identity between the test set and the training set of SARD80 and SVFD80 less than 80%. The benchmark model also includes FunGeneTyper-Random, which has the same structure and training methods as FunGeneTyper, but randomly initializes the parameters without loading the parameters of the pre-trained model. Diamond is used as a baseline model based on sequence similarity. Specifically, Diamond uses the training set as a reference dataset, and against testing set to the training set with parameters: --more-sensitive -e 1e-5 --max-target-seqs 1, and retained results with identity greater than 70% for baseline comparison analysis.

**Method S4. Evaluation of FunGeneTyper for the discovery of new functional genes**

To validate the capacity of FunGeneTyper models in discovering new functional genes, experimentally confirmed ARGs from functional metagenomics studies were retrieved from NCBI’s protein database (accession numbers in Dataset S8). We utilized Diamond to compare the collected sequences with the core ARG dataset and excluded any sequences that exhibited 100% identity match with the core ARG dataset. This exclusion was necessary because these sequences had already been used to train the model. After removing those ARGs that had a perfect sequence match to the CARD database (i.e., core dataset of ARGs), We retained a total of 297 experimentally confirmed ARG sequences from human gut[4] (n = 168), WWTPs[5] (n = 77), and soil[6-9] (n = 52) bacteria for downstream comparisons. These comparisons involved evaluating the classification performance of FunGeneTyper against the well-established SA-based (RGI[10]), HMM-based (Resfams[11]), and DL-based (DeepARG[12] and HMD-ARG[13]) approaches in terms of the new ARGs.

To compare the ability of FunGeneTyper for discovering new VFGs, BoNTs-like sequences from the genome of *Chryseobacterium piperi* reported in a prior study [14] was downloaded from NCBI’s database by accession number (Dataset S7). Then, VFGTyper was used to predict VFGs and their affiliated family from the BoNTs-like sequences, and the output results were compared with those by a conventional sequence alignment-based approach with Diamond[3] (version 2.0.15) search of the BoNTs-like sequences against SVFD.

**Method S5. Construction of structured virulence factor database (SVFD)**

*Core VFGs dataset.* Virulence factor databases were collected from VFNet(http://www.mgc.ac.cn/VFNet/) [1]. Zheng et al[1] performed a detailed similarity search for known and potential VFGs in the complete bacterial genome downloaded from the NCBI server using VFanalyzer[15], with Virulence Factor Database (VFDB) as the core database[15]. VFNet is an expanded virulence factor database that can be used directly in the training process. A two-level expert-curated structured database was established based on the virulence ontology and reference sequences in the VFNet database.

*Negative dataset.* The non-VFG (sequence counts: 471,009) collection process is similar to that of the non-ARG collection process, except that KW-0800 is used to filter sequences from Swiss-Prot database (version: June 2021).

# Supplementary Figures

Figure S1. Diagram of dataset construction for FunTrans and FunRep. FunTrans process on the left, FunRep process on the right. For the FunTrans model, the structured dataset is partitioned into training, validation and testing sets in a ratio of 6:2:2. For the FunRep model, only the core and expanded datasets from the structured database are utilized for fine-tuning training. Specifically, the entire core dataset is employed as the training dataset, and the expanded dataset is further divided into training, validation, and testing sets following a ratio of 6:2:2.

Figure S2. Diagrammatic representation of FunTrans and FunRep training architectures. a, The input data for FunTrans training is at the type level of the structured functional gene dataset (SFGD), containing negative examples of non-target sequences and positive examples labeled with functional types. FunTrans architecture are built upon a pretraining protein language model with integrated Adapter layers and incorporates an extra classification layer. b, The input data for FunTrans training is at the subtype level of the structured functional gene dataset (SFGD), including anchor sequences, positive sequences, and negative sequences. FunRep architecture is constructed on a pretraining protein language model with integrated Adapter layers. Following fine-tuning training, FunTrans and FunRep models are combined in series to assemble a complete FunGeneTyper model (e.g., ARGTyper).

Figure S3. The identity threshold for negative dataset expansion. Four non-target sequence sets collected from four progressively stricter identity thresholds as the negative datasets.

Figure S4. The number of ARGs class sequences in the core SARD. The x-axis is ARG class in core SARD. The y-axis is number of ARGs class sequences in the core SARD. SARD: structured ARG database.

Figure S5. Baseline testing and sequence similarity ablation analysis of SARD. A. Baseline test results on the original SARD dataset. B. In the baseline test of SARD data set with 80%identity similarity, ARGTyper can still effectively learn the features of sequences after removing similar sequences in the training set. SARD: structured ARG database.

Figure S6. Visualization of feature learning at the layer 33 in ARGs FunRep training. Each point is a two-dimensional mapping of a sequence of higher-dimensional representations. The same color indicatesthe same ARGs group. UMAP analysis shows the clustering of features in the same ARGs group.

Figure S7. An overview of the alignment of experimentally confirmed protein sequences. The identity distribution of alignment between the experimentally confirmed sequence and the core ARGs dataset. Experimentally confirmed protein sequences were divided into relatively lower homology (≤50% identity) and relatively higher homology (≥50% identity) datasets.

Figure S8. Confusion matrix for VFGs family level annotations. Confusion matrix for VFGs family classification, confusion between true (y-axis) and predicted (x-axis) VFGs. VFGs: virulence factor genes.

Figure S9. Baseline testing and sequence similarity ablation analysis of SVFD. **a**. Baseline test results on the original SVFD dataset. **b**. In the baseline test of SVFD data set with 80%identity similarity, VFGTyper can still effectively learn the features of sequences after removing similar sequences in the training set. SVFD: structured VFG database.

Figure S10. Visualization of feature learning at the layer 33 in FunRep training of VFGs. Each point is a two-dimensional mapping of a sequence of higher-dimensional representations. The same color indicates the same VFGs class. UMAP analysis shows the clustering of features in the same VFGs class.

**Reference:**

1. Zheng D, Pang G, Liu B et al. Learning transferable deep convolutional neural networks for the classification of bacterial virulence factors, Bioinformatics 2020;36:3693-3702.

2. Li W, Godzik A. Cd-hit: a fast program for clustering and comparing large sets of protein or nucleotide sequences, Bioinformatics 2006;22:1658-1659.

3. Buchfink B, Xie C, Huson DH. Fast and sensitive protein alignment using DIAMOND, Nat Methods 2015;12:59-60.

4. Sommer MOA, Dantas G, Church GM. Functional characterization of the antibiotic resistance reservoir in the human microflora, science 2009;325:1128-1131.

5. Munck C, Albertsen M, Telke A et al. Limited dissemination of the wastewater treatment plant core resistome, Nat Commun 2015;6:8452.

6. Willms IM, Grote M, Kocaturk M et al. Novel Soil-Derived Beta-Lactam, Chloramphenicol, Fosfomycin and Trimethoprim Resistance Genes Revealed by Functional Metagenomics, Antibiotics (Basel) 2021;10.

7. Wang S, Gao X, Gao Y et al. Tetracycline Resistance Genes Identified from Distinct Soil Environments in China by Functional Metagenomics, Front Microbiol 2017;8:1406.

8. Allen HK, Moe LA, Rodbumrer J et al. Functional metagenomics reveals diverse beta-lactamases in a remote Alaskan soil, ISME J 2009;3:243-251.

9. Donato JJ, Moe LA, Converse BJ et al. Metagenomic analysis of apple orchard soil reveals antibiotic resistance genes encoding predicted bifunctional proteins, Appl Environ Microbiol 2010;76:4396-4401.

10. Alcock BP, Raphenya AR, Lau TTY et al. CARD 2020: antibiotic resistome surveillance with the comprehensive antibiotic resistance database, Nucleic Acids Res 2020;48:D517-D525.

11. Gibson MK, Forsberg KJ, Dantas G. Improved annotation of antibiotic resistance determinants reveals microbial resistomes cluster by ecology, ISME J 2015;9:207-216.

12. Arango-Argoty G, Garner E, Pruden A et al. DeepARG: a deep learning approach for predicting antibiotic resistance genes from metagenomic data, Microbiome 2018;6:23.

13. Li Y, Xu Z, Han W et al. HMD-ARG: hierarchical multi-task deep learning for annotating antibiotic resistance genes, Microbiome 2021;9:40.

14. Mansfield MJ, Wentz TG, Zhang S et al. Bioinformatic discovery of a toxin family in Chryseobacterium piperi with sequence similarity to botulinum neurotoxins, Sci Rep 2019;9:1634.

15. Liu B, Zheng D, Jin Q et al. VFDB 2019: a comparative pathogenomic platform with an interactive web interface, Nucleic Acids Res 2019;47:D687-D692.
